# Does touch massage facilitate recovery after stroke? A study protocol of a randomized controlled trial

**DOI:** 10.1186/s12906-016-1029-9

**Published:** 2016-02-04

**Authors:** Kristina Lämås, Charlotte Häger, Lenita Lindgren, Per Wester, Christine Brulin

**Affiliations:** 1 Department of Nursing, Umeå University, Umeå, Sweden; 2 Department of Community Medicine and Rehabilitation, Umeå University, Umeå, Sweden; 3 Department of Public Health and Clinical Medicine, Division of Medicine at Umea University, Umea, Sweden

## Abstract

**Background:**

Despite high quality stroke care, decreased sensorimotor function, anxiety and pain often remain one year after stroke which can lead to impaired health and dependence, as well as higher healthcare costs. Touch massage (TM) has been proven to decrease anxiety and pain, and improve quality of health in other conditions of reduced health, where reduced anxiety seems to be the most pronounced benefit. Thus there are reasons to believe that TM may also reduce anxiety and pain, and improve quality of life after stroke. Further, several studies indicate that somatosensory stimulation can increase sensorimotor function, and it seems feasible to believe that TM could increase independence after stroke. In this study we will evaluate effects of TM after stroke compared to sham treatment.

**Methods:**

This is a prospective randomized open-labelled control trial with blinded evaluation (PROBE-design). Fifty patients with stroke admitted to stroke units will be randomized (1:1) to either a TM intervention or a non-active transcutaneous electrical nerve stimulation (non-TENS) control group. Ten sessions of 30 min treatments (TM or control) will be administered during two weeks. Assessment of status according to the International Classification of Functioning, Disability and Health (ICF), including body function, activity, and participation. Assessment of body function will include anxiety, pain, and stress response (heart rate variability and salivary cortisol), where anxiety is the primary outcome. Activity will be assessed by means of sensorimotor function and disability, and participation by means of health-related quality of life. Assessments will be made at baseline, after one week of treatment, after two weeks of treatment, and finally a follow-up after two months. The trial has been approved by the Regional Ethical Review Board.

**Discussion:**

TM seems to decrease anxiety and pain, increase health-related quality of life, and improve sensorimotor functions after stroke, but the field is largely unexplored. Considering the documented pleasant effects of massage in general, absence of reported adverse effects, and potential effects in relation to stroke, it is essential to evaluate effects of TM during the sub-acute phase after stroke. The results of this project will hopefully provide important knowledge for evidence-based care.

**Trial registration:**

ClinicalTrials.gov: NTC01883947

## Background

In Sweden, about 25,000 individuals suffer a stroke each year. Despite high quality stroke care in Sweden, decreased sensorimotor function, anxiety and pain often remain one year after stroke which may lead to impaired health and dependence, as well as to higher healthcare costs. It is therefore urgent to find new rehabilitation strategies. Touch massage (TM) is a massage treatment consisting of slow strokes with gentle pressure [1]. Although research indicates positive effects of TM on the wellbeing of healthy individuals and patients with ill-health conditions, no study has evaluated TM after stroke. Health is influenced in many ways after stroke, and in this study we focus on health components included in the International Classification of Functioning, Disability and Health (ICF) [2].

One component of ICF is Body Function and we focus in this study on anxiety, pain, and stress reactions. Concerning anxiety, about 50 % of stroke survivors experience anxiety in the acute stage of stroke [3]. In a Cochrane review [4], the authors concluded that there is insufficient evidence to make recommendations about treatment, and that more studies are needed. Our research group found that anxiety could be decreased following administration of TM in other ill-health conditions [1], a conclusion that has been confirmed in other studies (eg. [5–7]). In addition, using fMRI [8] we found increased neural activity in brain regions associated with feelings of pleasure and emotional regulation [9]. Headache, shoulder pain in the paretic shoulder, joint pain, and central post-stroke pain are common after stroke [10]. In earlier studies, our group found that TM can decrease the experience of pain [11], which is in line with results from other studies [12, 13]. To our knowledge, there is only one study [14] that has found an amelioration of pain and anxiety after slow back massage among patients with stroke in a rehabilitation unit. There are therefore good reasons to further explore this issue.

Stress reactions are common in patients suffering from stroke, and it is suggested that massage decreases the stress response and induces relaxation. The physiological mechanism, though, is not fully understood and contradictory results are reported. In a previous study, our research group did not find any significant changes in stress outcomes after TM (blood pressure, cortisol) [1], which may have been due to the small sample size. However, in a meta-analysis, Moraska and colleagues [15] found that eight of nine studies showed immediately decreased salivary cortisol levels after the first massage session. After several massage sessions, though, only five of nine studies reported decreased cortisol levels. In another meta-analysis [16] decrease in cortisol was found to be non-significant. Concerning blood pressure (BP), some studies report no change (e.g. [17]), while some indicate a decrease in systolic pressure (e.g. [18]) and others also reveal a decrease in diastolic pressure (e.g. [19]). In our pre-testing of massage on patients with stroke, we detected a decrease in systolic BP. Using heart rate variability (HRV), which reflects activity in the autonomic nervous system, we have previously found a decrease in sympathetic nervous activity with a subsequent compensatory decrease in parasympathetic nervous activity in healthy individuals receiving massage [20]. This, however, contrasts with other findings of an increase in parasympathetic activity during massage (e.g. [21]). Taken together, this presents a disparate picture regarding the physiological response to massage and more research is needed.

The second part of this project focuses on activity as another component of health in ICF. Sensorimotor function deficits are common after stroke, and depending on the lesions’ location and extent, the frequency of deficits will vary. Somatosensory deficits may cause difficulty managing daily living and prevent rehabilitation [22, 23]. In training somatosensory function after stroke, both improved sensory and motor function have been found [23]. Further, if somatosensory information is suppressed in healthy people, motor function is found to be impaired [24]. Thus, there appears to be strong support for the integration of sensory and motor function. There are both active and passive treatments related to sensorimotor function. Examples of active training are mirror therapy, repetitive sensory discrimination tasks, and sensory recognition training [22]. Other studies evaluate a more passive form of treatment and focus on sensory stimulation by electric stimulation, thermal stimulation, compression, magnetic stimulation [22] or acupuncture [25]. Thus, even if single studies report positive results, as the authors in a Cochrane review [22] conclude, there is insufficient evidence regarding the effectiveness of treatment for sensory loss after stroke, and more studies are needed. Some studies have reported that touch stimulation improves neurological development in children [26]. In the elderly, passive tactile stimulation has been shown to enhance tactile acuity, increase haptic object exploration skills and fine motor function [27]. Pre-clinical studies have shown that touch increased dendrite length and motor function [28], as well as increased neural activity and reperfusion in the involved area [29] in rats after stroke. One study of humans reported that mechanical touch influenced motor function after stroke [30] and thereby sensory stimulation is supposed to increase motor function recovery. Further, combining somatosensory and motor training post stroke (after 6 months or later) has been found to increase fine motor function, sensory discrimination, and musculoskeletal performance [31]. The increased function remained at follow-up three months later. To our knowledge, no study has investigated the effects of TM on stroke patients, although clinical experience shows that TM influences experiences of increased sensory function after stroke.

Stroke causes neurological deficits and it is therefore important to find interventions during the rehabilitation phase that can regain the patient’s abilities and promote neuronal recovery or compensation. Grefkes & Flink [32] argue that recovery from stroke is driven by neuronal reorganization in the brain. Studies using fMRI show that movements in the paretic limb induce abnormal activity in motor-related areas (both contralesional and ipsilesional hemispheres) compared to more focused activity in motor-related areas (contralateral hemisphere) in healthy controls. Furthermore, longitudinal studies have found that in subjects with good recovery, abnormal activity in motor-related areas is decreased and reaches levels similar to those found in healthy controls [32]. Some studies have evaluated brain activity in relation to motor rehabilitation after stroke [33]. The findings showed that in those who successful recovered following treatment, there was a shift from brain activity mainly in the contralesional motor cortex to activity mainly in the ipsilesional motor cortex when activating the affected limb. Also, stimulation of other senses after stroke, like visual stimulation by mirror therapy, affected the reorganization of brain activity toward the ipsilesional hemisphere [34]. The group participating in mirror therapy also improved according to the Fugl-Meyer motor assessment [34]. Results from the aforementioned studies suggest that successful treatment may normalize brain activity. However, as far as we know, no study has evaluated recovery after somatosensory stimulation by TM with respect to brain activity in stroke patients. It has been found, however, that sensory stimulation in stroke patients increase motor recovery, although the mechanisms are unknown [23].

Despite the often high quality of stroke care, decreased general health and independence are frequent in stroke survivors, and there is a need to further improve treatment and rehabilitation. Based on earlier research there are reasons to believe that TM may decrease anxiety, pain, stress reactions, and effect sensorimotor function after stroke and thus has the potential to improve everyday life after stroke. There is therefore reason to further evaluate the effects of TM after stroke.

### Aim, hypotheses and primary and secondary outcomes

This study will evaluate possible effects of TM after stroke on health in the ICF domains. Specific purposes are to investigate the impact on:

1. Body function by means of
  - Anxiety
  - Pain
  - Stress reactions
2. Activities by means of
  - Sensorimotor function
  - Disability
3. Participation by means of
  - Health-related quality of life

Our hypotheses are that TM after stroke will;

- decrease anxiety and pain (hypothesis I)
- decrease physiological stress responses (hypothesis II)
- increase sensorimotor function, which will be reflected in improved gross and fine motor skills and touch discrimination (hypothesis III)
- increase ability to perform activities in daily life (hypothesis IV)
- increase activity in contralateral sensorimotor areas and decrease redundant brain activity in motor-related areas. This will lead to more focused activity in motor-related areas, thus improving behavioural movement in the patients (hypothesis V)
- increase health-related quality of life (HRQoL) (hypothesis VI)

The primary outcome is change in anxiety measured by STAI. The secondary outcomes include pain, physiological stress responses by change in heart rate variability and salivary cortisol, gross and fine motor skills, grip strength, touch discrimination, quality in motor performance, activities in daily life, brain activity, and HRQoL (Table 1).

**Table 1:** Outcome assessments in relation to hypothesis and assessment methods

Outcome area	Hypothesis	Outcome	Assessment method
Body function	Hypothesis I	Anxiety (primary endpoint)	The State-Trait Anxiety Scale, (STAI)
		Pain, intensity	VAS
	Hypothesis II	Heart rate variability	ECG
		Salivary cortisol	
		Blood pressure	
Activities	Hypothesis III	Touch discrimination (sensory skills)	Shape Texture Identification Test (STI)
		Gross dexterity (motor skills)	Box and Blocks
		Fine motor dexterity (motor skills)	Nine Hole Peg
		Grip strength (motor skills)	Jamar® Hydraulic Hand Dynamometer
	(Subsample II)	Quality in motor performance	High-speed cameras in Movement laboratory
	Hypothesis IV	Disability after stroke	Modified Rankin Scale, Barthel index
	Hypothesis V (subsample 1)	Brain activity during finger-tapping/movement	GE 3 T scanner (General Electric, USA).
Participation	Hypothesis VI	Health-related quality of life	Nottingham Health Profile (NHP)

## Methods

### Design

This project is a prospective randomized open-labelled control trial with blinded evaluation (PROBE-design) of TM after stroke. Minimization will be used to create groups for intervention and control. Using minimization offers a way to stratify groups and ensure balance with respect to significant variables also in smaller samples. Minimization is considered to have an advantage in small trials (cf. [35]) and is an acceptable alternative to random assignment according to the CONSORT statement (http://www.consort-statement.org) and SPIRIT checklist (http://www.spirit-statement.org/spirit-statement/).

## Participants

### Participants

#### Inclusion criteria

Fifty participants will be recruited from two stroke units in northern Sweden within one week after the onset of stroke. The inclusion criteria will be 1) acute stroke defined according to the WHO-criteria of a neurological deficit of cerebrovascular cause that persists beyond 24 h [36]. The stroke cases are classified according to the TOAST (Trial of Org 10172 in Acute Stroke Treatment) [37] and OCSP (Oxford Community Stroke Project) [38] systems, 2) impaired finger tapping on the affected side of the body, 3) visible volitional flexion of the fingers on affected side of the body.

#### Exclusion criteria

Individuals with 1) cancer, 2) infections with fever, 3) psychiatric and 4) neurologic diseases and 5) alcohol or drug addiction may have affected general condition that will complicate interpretation of outcomes and will be excluded. 6) Conditions that impede communication can hamper the possibility to receive information and assess outcomes, therefore those individuals will also be excluded. A research nurse will collect information from the patients and staff concerning inclusion and exclusion criteria.

#### Power analysis and group distribution

In all, this study will include a total of 50 participants with 25 participants in each group. The power analysis is based on the primary outcome anxiety assessed with the State-Trait Anxiety Inventory (STAI) instrument (total score 20 – 80). Based on results from a previous study [1], the assumption is to find a difference in score corresponding to 8. Twelve subjects in each group will have an 80 % probability of achieving a statistically significant difference at a 5 % level. The subjects are allocated to the groups consecutively and by using minimization the groups are balanced with respect to severity in anxiety assessed with State-trait Anxiety Inventory (STAI) [39] and disability assessed with a Modified Rankin Scale (mRS).

Sub-sample I will consist of 30 participants (15 from the intervention group and 15 from the control group) who will be invited to participate in an fMRI study to evaluate brain activity during motor task performance. The inclusion criteria will be unilateral hemispheric stroke, intracerebral ischemic or hemorrhage lesion, first-ever stroke and cortical and/or subcortical lesions verified by radiology and/or clinical diagnosis, and fulfill the criteria for fMRI used at the Umeå Center for Functional Brain Imaging (UFBI), Umea University. An earlier study [40] found that the sample is adequate to produce stable and replicable results.

For feasibility purposes, a sub-sample (II) of 10 participants will be tested for dexterity in a movement laboratory before and after intervention.

### Recruitment procedure

A research nurse at the stroke unit will monitor admitted patients via patient records according to the inclusion and exclusion criteria and together with a stroke physician decide whether the patient is suitable or not. With a computer program the stroke physician will allocate the participant to one of the two groups and report group affiliation to the massage therapist. The massage therapist will give the patient oral and written information about the study and informed consent will be obtained.

### Intervention

In addition to ordinary care at the stroke unit, the interventions will be given by massage therapists experienced in TM. TM is a gentle massage with strokes on hands, arms, feet and legs with a pressure of 2.5 N which is more gentle than Swedish massage but harder than strokes performed with a soft brush. The speed of the strokes is about 1–5 cm/sec [20]. Hand massage is described by Snyder and co-workers [41]. During the massage, the subjects will lie on a bed in the ward, or if discharged, in their home. During treatment, no conversations will be initiated by the massage therapist. To assess if the massage intervention is effective beyond effects of expectation a sham treatment will be given to the subjects in the control group. The sham-treatment is a non-active transcutaneous electrical nerve stimulation (non-TENS), given while participants lie in bed with electrodes attached to the skin of the affected arm. The device will be manipulated in a way so that no electrical impulses will reach the electrodes. During treatment, the masseur will remain in the room without initiating any conversation. The treatments for both groups will start one week after the onset of stroke and last for 30 min each time, five days a week for two weeks. The choice to use non-TENS as a sham treatment is based on the fact that TENS has some commonalities to massage; TENS is a form of sensory stimulation, although different to massage, and can be applied to the same limbs that are treated in the massage treatment.

In case of progressive stroke symptoms or discomfort from the massage treatment the stroke physician will be contacted and the appropriateness of continuing the study will be discussed. Reasons for withdrawals will be recorded.

Since neither clinical experience nor research evidence give reasons to believe that the interventions risk patients’ safety, a data monitoring committee is not considered to be needed.

### Assessments

**Fig. 1:**
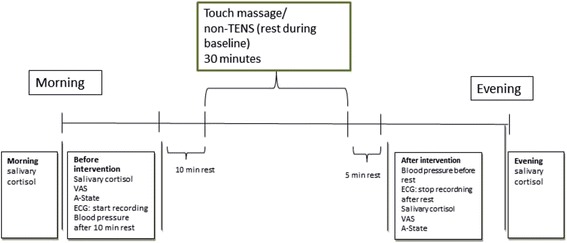
Flow chart including intervention and assessments of anxiety, pain and physiological parameters during a day, which will be repeated at BL, W1 and W2

**Fig. 2:**
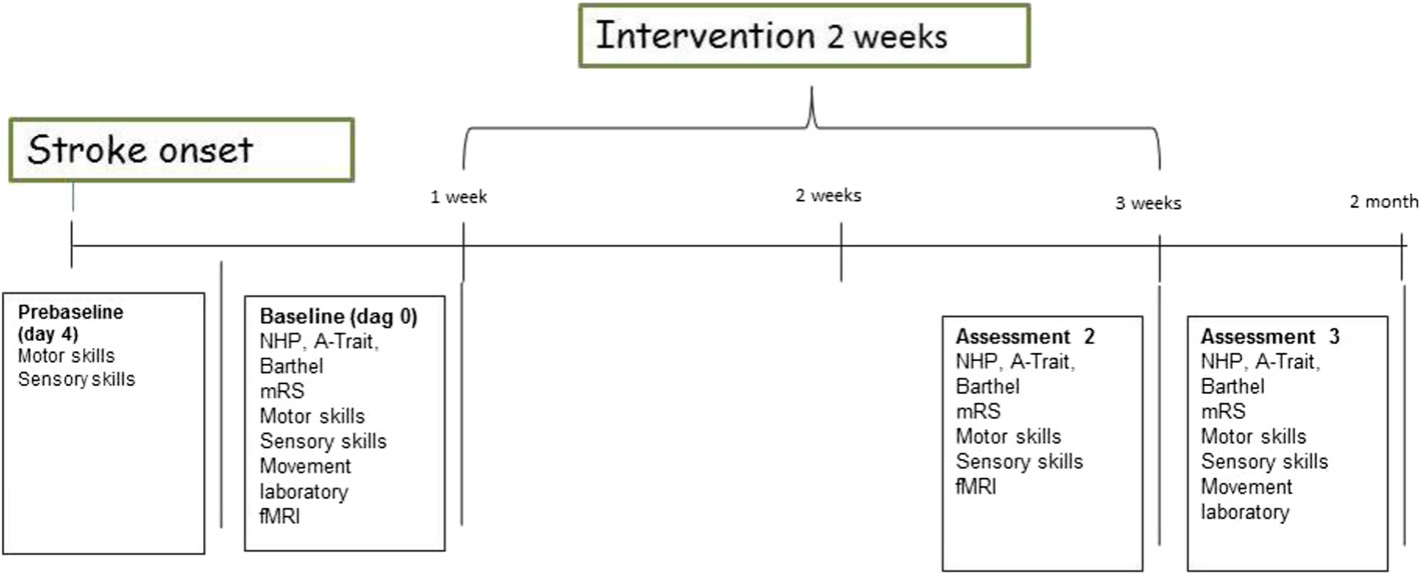
Flow chart including interventions and assessments related to sensorimotor function and health-related quality of life

Some of the effects are expected to occur immediately after a TM session and will be assessed before, during, and after the intervention session by the massage therapist (Fig. 1) on three occasions: baseline (BL), after one week (W1), and after two weeks (W2). Other effects are expected to occur over time and will be assessed on four occasions by a blinded assessor without knowledge of group affiliation. One occasion is a pre-baseline assessment in order to exclude the possibility that differences between baseline and follow up are merely a training effect. Long-term effects will thus be assessed at pre-baseline (pBL), baseline (BL), and follow-up 2 months after baseline (FU) (Fig. 2).

A summary of outcome assessments in relation to hypotheses and assessment methods can be found in Table 1.

For describing the study group, disability after stroke will be assessed by the Barthel index [42] and mRS [43]. In addition, age, gender, location of lesion, and acute treatment (thrombolysis/thrombectomy) will be recorded.

In this study we will attempt to assess components of body function such as anxiety, pain, and stress response. To test **hypothesis I** we will assess anxiety (primary endpoint) with the State-Trait Anxiety Inventory (STAI) [39]. Traits (A-Trait) are found to be stable over time and not sensitive to occasional stressors. These will be used at BL, W2, and FU. States (A-State) are found to be sensitive for occasional stressors assessing current emotional state [39] and will be used before and after treatment at BL, at W1 and W2. Pain will be assessed with a Visual Analogue Scale (VAS) [44]. To test **hypothesis II** (stress responses) we will record HRV as a continuous recording of a single-channel electrocardiogram (ECG) during TM at BL, W1 and W2. Salivary cortisol and blood pressure will be assessed in the morning, before and after TM and in the evening at BL, W1 and W2.

Further, we will assess effects on aspects related to activities as classified in ICF. Due to the limited amount of knowledge in this area and the heterogeneous sample, we will include a wide spectrum of assessments evaluating different aspects of activities. **Hypothesis III** (sensorimotor function) will be assessed with the Shape Texture Identification Test (STI) [45] to assess tactile gnosis, at PBL, BL, W2 and FU. STI has been found to have satisfactory validity and reliability [46, 47]. Box and Blocks test [48] will be used to test gross dexterity at PBL, BL, W2 and FU. The reliability and validity have been tested in several studies among different groups and found to be satisfactory [48]. To test fine motor dexterity, the Nine Hole Peg test [49] will be used at WBL, BL, W2 and FU. The Nine Hole Peg test has appropriate psychometric properties used among people following stroke [49]. The Jamar® Hydraulic Hand Dynamometer [50] will be used to assess grip strength, at WBL, BL, W2 and FU. Validity and reliability tests among healthy women have been satisfactory [51]. All sensorimotor tests will be performed based on standardized instructions with two trials for each hand starting with the unaffected hand. A movement laboratory with high-speed cameras will be used to perform advanced movement analysis with respect to quality in motor performance of hand and arm movement. Temporal and spatial kinematic variables will be evaluated. The participants will be evaluated pre- and post-intervention during functional manipulative tasks, such as the NineHole Peg test [49] and the Finger Nose test [52]. To test **hypothesis IV** the Barthel index and mRS will be used to assess ability after stroke at BL, W2 and FU. **Hypothesis V** will be tested in subsample 1 by evaluation of brain activity conducted on a GE 3 T scanner (General Electric, USA) in collaboration with the Umeå Center for Functional Brain Imaging (UFBI, Norrlands University hospital). The participants will perform finger movements with the paretic hand in the MR scanner while simultaneously recording the movements with special high-speed cameras, at BL and W2.

To assess effects on participation we will assess HRQoL with the Nottingham Health Profile (NHP) [53] which is a self-rating scale. NHP has been found to be a valid and sensitive measure of HRQoL [54] and is recommended to be used among people following stroke [55]. NHP will be used at BL, W2 and FU.

The assessments will be recorded on paper forms and the blinded assessor will provide each form with an ID code and store the code list in a locked cabinet. The first author will be responsible for the data entry of the coded paper forms.

### Data analysis

Data will be analyzed with repeated measurement methods to compare the intervention group and the control group. Statistical regression models that adjust for background characteristics and account for correlated measurements for the same individual will also be used (e.g., mixed effects model and generalized estimating equations). Data will be analyzed by inter-group analyses using the Statistical Package for Social Science (SPSS). Effect size will be estimated with Cohen’s d. The analysis will be performed on an intention-to-treat basis.

HRV will be analyzed by automatic detection of heartbeats, then the R-R interval data will be derived and transformed into an evenly sampled heart rate time series. From this time series HRV will be analysed in the frequency domain where the relative influence of different spectral components will be determined. From the signal, the mean heart rate, the total spectral power (PTOT), and the power of the low-frequency (PLF; 0.04-0.15 Hz) and high-frequency (PHF; 0.15-0.40 Hz) will be calculated. The recording and analysis software has been developed at Department of Biomedical Engineering (DBE), Vasterbottens county council (VLL) and will be analysed in collaboration with DBE, VLL.

The fMRI data will be analyzed in collaboration with experts at the UFBI. The effects of TM on sensorimotor functions and brain activity over time will be studied and compared within and between the intervention and control groups. The analysis will be similar to an earlier fMRI study [56].

### Ethical considerations

The study has been approved by the Regional Ethical Review board in Umeå (2012-110-31). Written informed consent to participate and to publish results will be obtained. The data will be coded and only the research group will have access to the data set and the code list. Double-blind randomization is difficult when assessing effects of massage, and it is common to compare the effects of massage with those during resting. In our study, we have chosen TENS without electric impulses as a sham treatment in order to rule out the possibility that the effects result from receiving any type of treatment whatsoever. The value of this approach is considered to outweigh any possible harm that might result from being subjected to sham treatment and feeling deceived. No physical harm is expected.

## Discussion

TM has been found to have beneficial impacts on a variety of conditions. It increases relaxation and wellbeing in healthy individuals, in the elderly, and in others with a variety of health conditions. It is also noteworthy that we, in an earlier project, found decreased anxiety and pain and increased wellbeing among patients, and further, increased brain activity in areas related to feelings of pleasure and emotional regulation among healthy volunteers. These findings provide reason to believe that TM reduces anxiety and pain, and improves quality of life after stroke. Furthermore, several studies indicate that somatosensory stimulation can increase sensorimotor function, and it is logical to believe that TM increases independence after stroke. Although clinical studies are crucial, they raise several challenges. In Sweden in the 1990′s, high expectations were raised about improved recovery following treatment with acupuncture after stroke. A small study showed promising results of acupuncture in relation to improved motor function and ADL when compared to ordinary care [25]. When repeated in a multicentre trial with two control groups with high intensity and low intensity TENS respectively, acupuncture was not proved to have beneficial effects [57]. In an overview of systematic reviews and meta-analyses [58, 59] effects of acupuncture on disability after stroke were not evident. However, there is some evidence for improved dysphagia and global neurological deficit score. The authors [58, 59] concluded that rigorous RCTs are needed to confirm the results. This illustrates the difficulties in conducting reliable clinical studies and it seems essential to include control groups that receive either a comparable or a non-active treatment to e.g. reduce the Hawthorn effect [60]. In our study we plan to have a control group that receives a non-active TENS treatment as argued for above. Also, sample size is crucial in order to produce reliable results. In our study the sample size is based on results from earlier research that found decreased anxiety after massage treatment in other health disorders. Anxiety is common after stroke and by reducing anxiety it is reasonable to expect that the patient is in a better position to manage the demanding rehabilitation phase. Effects of massage on sensorimotor function is more speculative but theoretically, based on earlier knowledge, it seems reasonable that massage could have positive effects on sensorimotor function. Our study can hopefully guide further well-grounded research within this area. In brief, the present study aims to provide a greater understanding of the effects of TM in rehabilitation after stroke and increase knowledge that will contribute to explanatory models for TM. It is a first step, which we hope will serve as an essential foundation for a future multicentre trial. The results of this project could potentially generate important knowledge for evidence-based care because of the current limited understanding of the effects of TM following stroke.

## Abbreviations

A-State: The state-trait anxiety scale, subscale state
A-Trait: The state-trait anxiety scale, subscale trait
BL: Baseline
BT: Blood pressure
DBE: Department of biomedical engineering
ECG: Electrocardiogram
fMRI: Functional magnetic resonance imaging
FU: Follow up 2 months after baseline
HRQoL: Health related quality of life
HRV: Heart rate variability
Hz: Hertz
MR: Magnetic resonance
mRS: Modified ranking scale
N: Newton
NHP: Nottingham health profile
OCSP: The oxford community stroke project classification
PBL: Pre baseline
PHF: Power of high-frequency
PLF: Power of low-frequency
PTOT: Total spectral power
SPSS: Statistical package for social science
STAI: State-trait anxiety inventory
TENS: Transcutaneous electrical nerve stimulation
TM: Touch massage
TOAST: Trial of org 10172 in acute stroke treatment
UFBI: Umeå center for functional brain imaging
VAS: Visual analogue scale
VLL: Västerbotten county council
W1: After one week intervention
W2: After two weeks intervention
